# ENhancinG vAGinal dElivery in Greece through educational and behavioral interventions among maternity care providers regarding labor management: the ENGAGE stepped-wedge randomized prospective trial protocol

**DOI:** 10.1186/s13063-024-08263-x

**Published:** 2024-08-19

**Authors:** Nikolaos Vrachnis, Nikolaos Antonakopoulos, Peter von Dadelszen, Marianne Vidler, Georgios Maroudias, Jeffrey Bone, Ash Sandhu, Nikolaos Loukas, Laura Magee, Nikolaos Roussos, Stefania Kassaris, Alexandros Fotiou, Dimitrios Zygouris, Georgios Adonakis, Christodoulos Akrivis, Aris Antsaklis, Apostolos Athanasiadis, Nikolaos Bontis, Angelos Daniilidis, Alexandros Daponte, Georgios Daskalakis, Efthimios Deligeoroglou, Konstantinos Dinas, Peter Drakakis, Angeliki Gerede, Grigorios Grimbizis, Nicoletta Iacovidou, Nikolaos Kambas, Theodoros Katasos, Christos Katsetos, Ilias Katsikis, Antonios Makrigiannakis, Michail Matalliotakis, Christina Messini, Themis Mikos, Nikolaos Nikolettos, Georgios Pados, Minas Paschopoulos, Konstantinos Patsouras, Soultana Siahanidou, Vasileios Sioulas, Chara Skentou, Sofoklis Stavros, Marleen Temmerman, Panagiotis Tsikouras, Vasilios Tsitsis, Nikolaos Vlahos, Alexandros Rodolakis, Aris Papageorghiou, Dimitrios Loutradis

**Affiliations:** 1 3rd Department of Obstetrics and Gynecology of the University of Athens, Attikon Hospital, Athens, Greece; 2 St George’s University Hospital NHS Foundation trust, London, UK; 3 Department of Obstetrics and Gynecology of the University of Patras, University Hospital of Patras, Patras, Greece; 4 Department of Women and Children’s Health, School of Life Course Sciences, Institute of Women and Children’s Health, King’s Health Partners Academic Health Science Centre, King’s College London, London, UK; 5 Women’s Health Research Institute/Medicine, Department of Maternal and Fetal Medicine & Pediatric Anesthesia, University of British Columbia, Vancouver, Canada; 6 Department of Obstetrics and Gynecology, Tzaneio General Hospital of Piraeus, Piraeus, Greece; 7 B.C. Women’s and Children’s Hospital, University of British Columbia, Vancouver, Canada; 8 King’s College London, London, UK; 9 1st Department of Obstetrics and Gynecology of the University of Thessaloniki, Papageorgiou Hospital, Thessaloniki, Greece; 10 Faculty of Medicine, University of British Columbia, Vancouver, Canada; 11 Gynecological Oncology Unit, St. Luke’s Hospital, Thessaloniki, Greece; 12 Department of Obstetrics and Gynecology, Chatzikosta Hospital, Ioannina, Greece; 13 Department of Obstetrics and Gynecology, Iaso Hospital, Athens, Greece; 14 3rd Department of Obstetrics and Gynecology of the University of Thessaloniki, Ippokratio Hospital, Thessaloniki, Greece; 15 Eleftho Obstetric Clinic, Kavala, Greece; 16 2nd Department of Obstetrics and Gynecology of the University of Thessaloniki, Ippokratio Hospital, Thessaloniki, Greece; 17 Department of Obstetrics and Gynecology of the University of Larissa, University Hospital of Larissa, Larissa, Greece; 18 1st Department of Obstetrics and Gynecology of the University of Athens, Alexandra Hospital, Athens, Greece; 19 2nd Department of Obstetrics and Gynecology of the University of Athens, Aretaieion Hospital, Athens, Greece; 20 Department of Obstetrics and Gynecology of the University of Thrace, University Hospital of Alexandroupolis, Alexandroupolis, Greece; 21 Department of Neonatology of the University of Athens, Aretaieion Hospital, Athens, Greece; 22 Department of Obstetrics and Gynecology, Hospital of Corinth, Corinth, Greece; 23 Department of Obstetrics and Gynecology, Hospital of Agios Nikolaos, Agios Nikolaos, Greece; 24 Department of Obstetrics and Gynecology, Viokliniki Hospital, Thessaloniki, Greece; 25 Department of Obstetrics and Gynecology of the University of Crete, University Hospital of Heraklion, Heraklion, Greece; 26 Department of Obstetrics and Gynecology, Venizeleio General Hospital of Heraklion, Heraklion, Greece; 27 Department of Obstetrics and Gynecology, Diavalkanikon Hospital, Thessaloniki, Greece; 28 Department of Obstetrics and Gynecology of the University of Ioannina, University Hospital of Ioannina, Ioannina, Greece; 29 Neonatal Unit, First Department of Pediatrics, Athens University Medical School, Athens, Greece; 30 Department of Obstetrics and Gynecology, Mitera Hospital, Athens, Greece; 31 Department of Obstetrics & Gynaecology, Aga Khan University Hospital, Nairobi, Kenya; 32 Department of Obstetrics and Gynecology, Hospital of Pyrgos, Pyrgos, Greece; 33 Nuffield Department of Women’s & Reproductive Health, University of Oxford, Oxford, UK

**Keywords:** Cesarean section, Vaginal delivery, Birth, Greece, Labor, Guideline, Maternal morbidity, Neonatal morbidity, Perinatal morbidity, ENGAGE trial, Stepped-wedge randomized trial, Robson classification, Intervention, Behavioral changes

## Abstract

**Background:**

There is an emerging need to systematically investigate the causes for the increased cesarean section rates in Greece and undertake interventions so as to substantially reduce its rates. To this end, the ability of the participating Greek obstetricians to follow evidence-based guidelines and respond to other educational and behavioral interventions while managing labor will be explored, along with barriers and enablers. Herein discussed is the protocol of a stepped-wedge designed intervention trial in Greek maternity units with the aforementioned goals in mind, named ENGAGE (ENhancinG vAGinal dElivery in Greece).

**Methods:**

Twenty-two selected maternity units in Greece will participate in a multicenter stepped-wedge randomized prospective trial involving 20,000 to 25,000 births, with two of them entering the intervention period of the study each month (stepped randomization). The maternity care units entering the study will apply the suggested interventions for a period of 8–18 months depending on the time they enter the intervention stage of the study. There will also be an initial phase of the study lasting from 8 to 18 months including observation and recording of the routine practice (cesarean section, vaginal birth, and maternal and perinatal morbidity and mortality) in the participating units. The second phase, the intervention period, will include such interventions as the application of the HSOG (the Hellenic Society of Obstetrics and Gynecology) Guidelines on labor management, training on the correct interpretation of cardiotocography, and dealing with emergencies in vaginal deliveries, while the steering committee members will be available to discuss and implement organizational and behavioral changes, answer questions, clarify relevant issues, and provide practical instructions to the participating healthcare professionals during regular visits or video conferences. Furthermore, during the study, the results will be available for the participating units in order for them to monitor their own performance while also receiving feedback regarding their rates. Τhe final 2-month phase of the study will be devoted to completing follow-up questionnaires with data concerning maternal and neonatal morbidities that occurred after the completion of the intervention period. The total duration of the study is estimated at 28 months. The primary outcome assessed will be the cesarean section rate change and the secondary outcomes will be maternal and neonatal morbidity and mortality.

**Discussion:**

The study is expected to yield new information on the effects, advantages, possibilities, and challenges of consistent clinical engagement and implementation of behavioral, educational, and organizational interventions described in detail in the protocol on cesarean section practice in Greece. The results may lead to new insights into means of improving the quality of maternal and neonatal care, particularly since this represents a shared effort to reduce the high cesarean section rates in Greece and, moreover, points the way to their reduction in other countries.

**Trial registration:**

NCT 04504500 (ClinicalTrials.gov). The trial was prospectively registered.

Ethics Reference No: 320/23.6.2020, Bioethics and Conduct Committee, School of Medicine, National and Kapodistrian University of Athens, Athens, Greece

**Supplementary Information:**

The online version contains supplementary material available at 10.1186/s13063-024-08263-x.

## Introduction

Since 1985, the international healthcare community has considered the ideal rate for cesarean sections to be between 10 and 15% [1]. However, cesarean sections have become increasingly common in both developed and developing countries so that today, the cesarean section rate worldwide is much higher than the rate proposed by the World Health Organization (WHO) [2]. While medically required cesarean section is associated with lower maternal and neonatal mortality, it has not been demonstrated to have any benefit for mothers and offspring who do not need the procedure. Statistically speaking, data from the previous decade show that when cesarean section rates rise above 10%, there is no maternal mortality rate improvement. A cesarean section is surgery, and, as in any surgery, short- and long-term risks may be involved which can have serious after-effects for years afterwards: following the delivery, the health of the woman, her offspring child, and future pregnancies may be compromised. Such a potential is greater among women who have little to no access to full pregnancy care. For this reason, governments and clinicians have over the past few years voiced concern regarding the steep increase in cesarean section births and, thus, the possibility of long-term negative effects for both mother and child [3]. Of course, vaginal delivery, especially operative vaginal delivery can also be associated with risks and adverse events, such as failure to progress, abnormal fetal heart rate pattern, intrapartum and postpartum hemorrhage, severe perineal lacerations, pelvic floor disorders, postpartum sexual dysfunction, and fetal injuries, certain conditions require immediate conversion of vaginal delivery to an emergency cesarean section [4].

In Greece, there is a lack of accurate statistical data on the cesarean section rate, but it seems to be above 50% [5–8], with many clinicians not availing themselves of the alternatives of vaginal birth practices proven to be beneficial [9]. In 2014, HSOG (the Hellenic Society of Obstetrics and Gynecology) published a position statement expressing concerns on the high cesarean section rates in Greece. The question as to why harmful and/or unnecessary procedures are used in many cases while other beneficial practices are ignored, despite active dissemination of scientific evidence still remains largely unanswered. The fact is that the explanation for the increased cesarean section rate is not readily apparent as it is the outcome of multiple contributing factors, iatrogenic and non-iatrogenic: these include non-adherence to the international obstetric guidelines, the lack of cesarean section audits and a serious shortage of midwives [10]. Primary cesarean section can be characterized as a key factor in the overall increase of cesarean sections given the vicious cycle of recurrence of a cesarean delivery. Hence, once there has been a reduction in the factors that lead to primary cesarean section, we can expect to see a drop in cesarean section rates. It is therefore clear that each case must be individualized and carefully evaluated as to whether or not it meets the criteria based on the guidelines for cesarean delivery [11, 12].

The fact that we currently have a poor understanding of this entire situation is partly due to the absence of a standardized internationally-accepted classification system that would monitor and compare cesarean section rates in a uniform and concrete manner. To this end, the WHO has proposed the international adoption and application of the Robson classification system for the classification of cesarean sections [1]. A theoretical framework would target specific interventions to address appropriate practice patterns after delineating the barriers to the adoption of evidence-based birth practices [13, 14]. These barriers mainly include lack of knowledge or awareness, inadequate familiarity with the issue, failure to keep up with the literature, as well as such attitude issues as the inertia of previous practices, outcome expectancy, and fear of litigation for “substandard” obstetric care and “low” self-efficacy. Other external factors affecting behavior are also noted, including lack of resources, poor time management, and doubts concerning ease of process.

Key to enabling enhancement of awareness of and familiarity with best clinical practice will be rendering research information accessible and comprehensible to practitioners. The application of evidence-based guidelines on labor management is an appropriate way to deal with the mode of delivery queries so as to reduce the cesarean section rates and, thus, to substantially improve maternal and neonatal health care thereby lowering the respective morbidity rates [15–19]. Since there is no “magic formula” to change professional behavior, the best approach may be to combine several strategies focusing on the same target, such as clarifying local leaders’ opinions, convening workshops, providing educational outreach visits, including lectures and interactive case discussions, and providing charts and other reminders along with audit procedures with provision for feedback [20–26]. These additional interventions while applied during our trial are expected to contribute further and they are discussed later in the text.

We hypothesize that a multifaceted intervention that is devised to enlighten and educate birth care providers and attendants as to the efficacy of previously applied birth practices will provide them with the theoretical background and skills to interpret evidence-based birth practices and modules and to sustain them over time, which will eventually lead to reduced cesarean section rates. The study consists of two phases, namely, observational and interventional. It will assess current practice in the participating obstetric departments and the implementation of interventions. Our aim is to determine whether this set of interventions will lead to lower cesarean section rates as well as to explore any impact on maternal and neonatal morbidity. As far as we know, this will be the first randomized, controlled, prospective trial carried out to assess the effectiveness of applying specific interventions in order to implement evidence-based birth practices with the aim of reducing the unjustifiably high cesarean section rates.

## Methods

### Study design

**Fig. 1:**
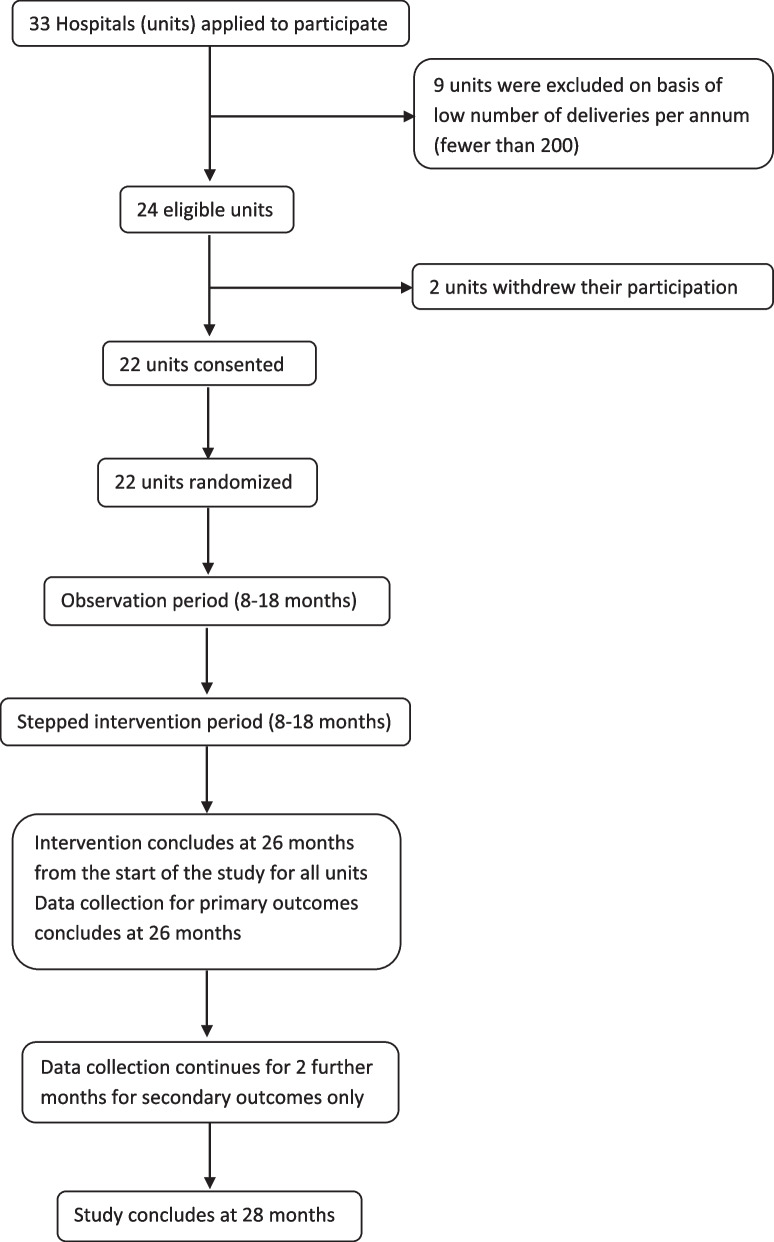
Study flowchart

The plan is to conduct a multicenter stepped-wedge randomized trial involving approximately 20,000–25,000 births in selected maternity care centers around Greece. The participating obstetrical units will be located in Athens and other cities throughout the country, each one reflecting its local population characteristics, whether similar or different as compared to each other. The study will include both public (National Health System (NHS) and university) and private maternity units for the purpose of assessing different types of clinical settings. An invitation for participation was sent out by the HSOG, and the units that applied with the most deliveries among the three categories (National Health System, university, and private) were included. Eligibility of each clinic to participate in the study was backed by a minimum of 5 years’ provision of obstetric services prior to intervention initiation, considering this time period necessary and sufficient for a standard operating level of the unit. In total, 22 obstetrical units will be entering the study, as shown in the study flowchart (Fig. 1, Table 1).

**Table 1:** Participating units

	**Ηospital/unit**
1.	Alexandra University Hospital, Athens
2.	Papageorgiou University Hospital, Thessaloniki
3.	University Hospital, Larissa
4.	University Hospital, Ioannina
5.	Ippokratio University Hospital, Thessaloniki/3^rd^ department
6.	Ippokratio University Hospital, Thessaloniki/2^nd^ department
7.	Attikon University Hospital, Athens
8.	University Hospital, Patras
9.	Aretaieion University Hospital, Athens
10.	University Hospital, Heraklion
11.	University Hospital, Alexandroupolis
12.	NHS Hospital “Venizeleio”, Heraklion
13.	NHS Hospital “Chatzikosta”, Ioannina
14.	NHS Hospital, Agios Nikolaos
15.	NHS Hospital, Pyrgos
16.	NHS Hospital, Corinth
17.	NHS Hospital “Tzaneio”, Piraeus
18.	Iaso Private Hospital, Athens
19.	Mitera Private Hospital, Athens
20.	Diavalkanikon Private Hospital, Thessaloniki
21.	Viokliniki Private Hospital, Thessaloniki
22.	Eleftho Private Hospital, Kavala

The participating units are recruited by the trial coordinators using geographic/population criteria and the number of births per year. The births occurring in each department and the participating obstetrician will be recruited in the study given that a maximum of 11 obstetricians per department will be involved, based on the number of births allocated to each professional. If a department has more than 11 obstetricians performing deliveries, obstetricians with the larger number of births will take part. This was decided in order to clearly define the data collection of the study. The only obstetricians or pregnant women excluded from the study will be those unwilling to participate. Informed consent of the pregnant women will be obtained by the enrolled obstetricians responsible for the care of each participating woman. The consent informs the woman about the details of the trial. The number of 11 participating obstetricians per site was determined by the 11 steps of the trial (11 pairs of units involved); thus, up to 11 obstetricians were allowed to participate. This restriction to the number is standard for stepped-wedge trials and is based on the number of steps. On the other hand, the majority of participating units had approximately this number of obstetricians or fewer. In units with more obstetricians, an effort was made to include those with the higher numbers of deliveries, irrespective of their vaginal birth or cesarean section rates.

The study does not include any interventions in the mothers or their fetuses and does not involve any pharmaceutical intervention or collection and handling of biological specimens. Instead, our study intervention applies only to the obstetricians and their labor ward practice. We will determine, among others, whether a cause of the increased cesarean section rates is a lack of compliance with current guidelines on labor management or the obstetricians’ tendency to favor a date and time convenient type of surgical labor management. In either case, obstetricians’ adherence to guidelines and follow-up tools for surgical management of labor is also expected to reduce the number of unnecessary cesarean sections or misguided interventions that can lead to cesarean section and change the doctor’s approach to vaginal birth.

The stepped-wedge cluster randomized trial comprises a novel research study design composed of the sequential transition of clusters (units) from control (observation) to intervention in a randomized order, until all clusters (units) have been revealed [27–30]. In current practice, this modality is increasingly being used for the evaluation of interventions. It will also reduce all practical and financial constraints to the minimum. Furthermore, there will be an effort to ensure that all enrolled maternity care professionals working in the selected units will comply with the intervention.

Specifically, there will be a period of monitoring and recording the activities of the maternity care unit with regard to the vaginal births and the cesarean sections (routine recording). During this period, professionals will continue with their usual in-service activities. The study will begin simultaneously in all maternity units, with a minimum recording period of routine activity for 8 months. During this period, the maternal and perinatal outcomes in each unit will also be recorded in detail via completion of questionnaires. The observation and intervention period durations will vary among the units although all units will enter the study at the same time; the intervention will be initiated in a different month (step wedged) for each of them. All the units will conclude the study at the same time, 28 months later.

During the study, there will be regular discussion of the results with the participating units. Moreover, a review of the local cesarean section rates and suggestions for improvement will take place at the start of the intervention period. We will also endeavor to ensure that all maternity care professionals working in the selected units are adequately informed about the guidelines and the other proposed interventions and have understood them. A meeting will take place shortly before the initiation of the intervention period, after which there will be online communication on a regular basis (once or twice per month). Adequate information will be provided to all the staff involved in the study and the unit’s director or designee/opinion leader whose responsibility will be to inform the other team members of the study results. A member of the study’s staff will visit each unit four times per year. During the visit she/he will accommodate data acquisition and local participating obstetricians’ familiarity with the study’s platform and will also serve the study committees’ activities indicated locally. These strategies are expected to improve adherence to the intervention. There will be no blinding for the care providers (obstetricians) concerning the results of their unit.

The intervention period of the study will be 8–18 months long and the participating units will be randomized centrally by the Clinical Trials Unit of the British Columbia University of Vancouver, Canada. According to the study design, two units will enter the intervention phase each month. This will last for 11 months. The data from each birth will be inserted electronically and will be accessible to each unit in real time. No modification of the allocated interventions is accepted without an apparent need and this should be clearly reported and explained to the study’s coordinators. No obstetrical intervention individually justified for each case will be affected by the trial protocol and the study cannot cause any harm to the participating mothers and their fetuses/newborns.

**Fig. 2:**
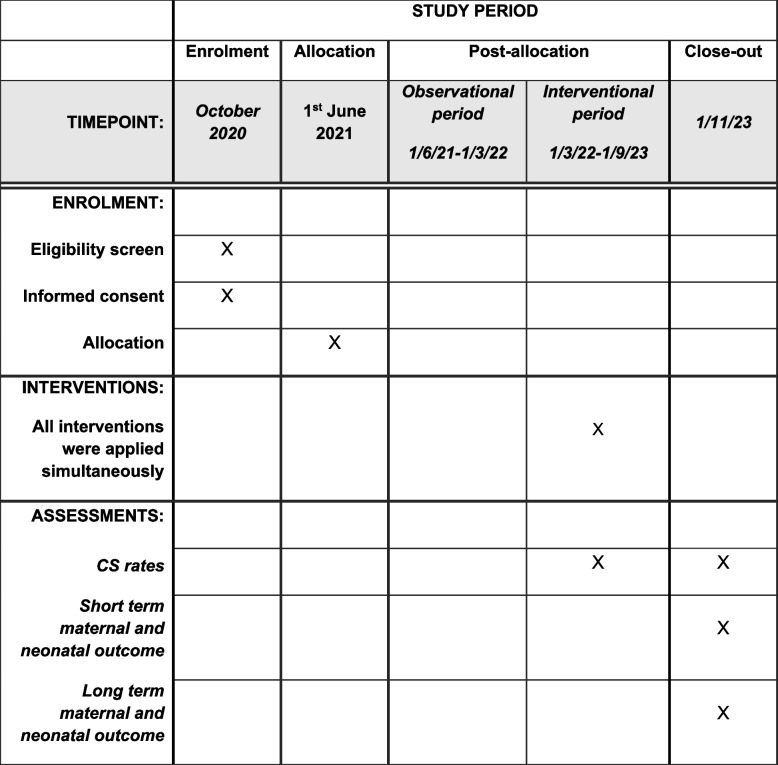
Schedule of enrolment, interventions, and assessments for ENGAGE trial

More specifically, the trial phases are as follows. The first phase involves the observation and recording of cesarean sections and vaginal births and relevant information of the participating maternity hospitals (routine practice recording) and starts synchronously for all units. The second phase, which is of different duration for each maternity hospital (stepped randomization), will comprise the implementation of the interventions following their presentation and discussion by the trial coordinators and local opinion leaders with the unit’s medical and midwifery staff. During each stage of communication, it will be stated explicitly that this implementation is being monitored and evaluated. During the study, relevant questions will be answered, and requested clarification will be provided to the healthcare professionals involved. Apart from the statistics provided in the online platform, staff involved in the study will receive regular newsletters on the progress of the study and will have the opportunity to interact with the study coordinators at least one time during the onsite visit. A two-month period for collection of the last telephone questionnaires will follow after the end of the intervention period. The total duration of the study is estimated at 28 months (8 months of observation, 18 months of stepped intervention, and 2 months collecting the last telephone questionnaires), as shown in Table 2 and Fig. 2.

**Table 2:**
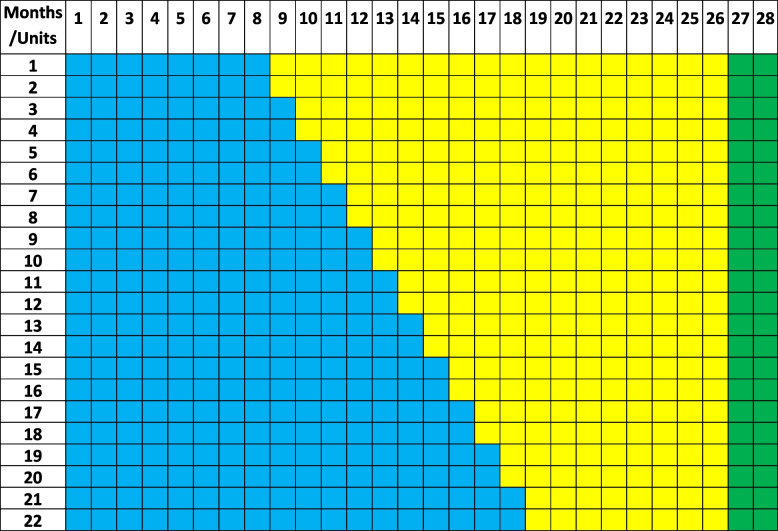
Timetable of the prospective stepped-wedge randomized controlled trial. The trial will terminate in 26 months from its initiation

Blue: Observation phase (months 1–8 or more)

Yellow: Intervention phase (months 9–26)

Green: Post-rollout period (maternal feedback by telephone of the last questionnaires, months 27–28)

### Interventions

The 18-month interventions will include a presentation of the HSOG Guidelines, case discussions focusing on guidelines application, training workshops for evidence-based best practices, use of a purpose-built website, educational reminders, and auditing and feedback on the utilization rates [31, 32]. There will be intervention at all the different levels of unit clinical practice: director, obstetricians, trainees in obstetrics, and midwives. Table 3 summarizes the components of the study’s intervention.

**Table 3:** Components of study’s intervention

**Local opinion leaders**
-Departmental chair and/or obstetrician nominated by departmental chair
**Electronic files sent to the participants** -PDF info and video: use of REDCap platform, presentation of study’s questionnaires, communicating study’s objectives (at the start of the observation period)
**Interactive webinars, courses, and workshops** -All staff involved in deliveries and collaborating with the participating obstetricians will join the following workshops: A. 2-day course, just prior to the intervention period. This course will be repeated 2–3 times for each unit during the intervention period. • Day 1 – Part 1: Theory - presentations and interactive discussion: - ENGAGE study interventions - HSOG Guideline on cesarean section and VBAC - HSOG Guideline on induction and augmentation of labor - Robson cesarean sections classification - Organization of labor ward - Obstetric emergencies - Medicolegal issues in cesarean sections • Day 1 – Part 2: Video demonstrations - Breech delivery - Twins delivery - External cephalic version - Delivery of other abnormal presentations - Forceps - VBAC - Fetal blood sampling • Day 2: Cardiotocography (CTG) physiological interpretation course (accurate evaluation of fetal distress and avoidance of unnecessary interventions) B. 1-day hands-on workshop on site: - Breech delivery - Twins delivery - External cephalic version - Delivery of other abnormal presentations - Forceps - VBAC C. 1-day meetings (recurring once or twice monthly): cesarean section and CTG online meetings with review of patient notes (duration 60 min)
**Detailing** -Advice to overcome barriers of different categories -Discussion with participating obstetricians and supporting staff during the intervention period about organization issues and adaptation to changes
**Instruction on achieving compliance with best practices** -Training within courses and meeting or teleconference with members of the steering committee when issues arise
**Compliance enhancement** -Regular meetings locally (cardiotocography meeting, cesarean section meeting, once or twice monthly, during the intervention period) -Follow-up meetings with study’s facilitators (physical presence, teleconferences every 2–4 weeks, phone calls) about the above topics or others which may arise -A dedicated person (member of study’s staff) will visit each unit every 3 months. During the visit she/he will accommodate data acquisition and local participating obstetricians’ familiarity with the study’s platform and serve the study’s board activities indicated locally.
**Reminders of good obstetric practice** -Placing reminders for enhancement of vaginal birth in labor wards. Placing posters in staff rooms and above theater hand wash basins. - Mobile phone short text messages - Newsletter messages sent on a weekly basis during the intervention period
**Audit and feedback** -Regular email reports of departmental performance. Live real-time statistics will be available for each participating department on the REDCap platform.
**Information technology** A website has been developed (www.emgetrial.gr) for the participating obstetricians and staff to access the guidelines and download evidence-based information (plus other information, e.g., Robson criteria for cesarean sections).

Various types of intervention will be provided:

1. The establishment in daily practice of three guidelines and two consent forms that were published by the HSOG. The titles of the guidelines and the consent forms are depicted in Table 4. Realizing the crucial need to apply evidence-based medicine in obstetrics, HSOG has published these guidelines and consent forms based on international guidelines. All of them were posted and made freely available for comments on the HSOG website (www.hsog.gr) for at least one month. Following this, further feedback was provided by the HSOG’s Scientific Committee, subsequent to which the guidelines were approved by the Society’s Board for final publication. The same procedure was followed for the two consent forms. The guidelines and consent forms are written in Greek and are freely available in PDF format through the Society’s website. The principal investigator and the local trial investigator discuss and disseminate the guidelines among the participating staff at the respective units, providing the opportunity to identify specific barriers of their practice. This requirement was instituted so that the planned activities could be adapted in such a way as to overcome them as well as to draw up an implementation timetable [33].
2. At least one session with the physical presence of the Principal Investigator, the trial coordinators, and the staff will take place shortly before they switch on the intervention period in each participating unit so that the local obstetricians and midwives may be informed on the objectives and interventions of the study. Prior to this, correspondence by email will provide details, such as the trial questionnaires and other required paperwork.
3. All staff, including midwives and trainee doctors, involved in the participating units will receive a PDF document and a video just prior to the study initiation. These explain the use of the purpose-built electronic platform (REDCap) and how to fill the study’s questionnaires.
4. All staff, including midwives and trainee doctors, involved in deliveries and collaborating with participating obstetricians will participate in interactive courses and workshops immediately prior to the intervention period of the study. There will also be a 2-day online course just prior the intervention period. The 1^st^ day’s schedule will include presentations and interactive discussion on such topics as ENGAGE study interventions, the HSOG Guideline on cesarean section and VBAC, the HSOG Guideline on induction and augmentation of labor, Robson cesarean sections classification, organization of the labor ward, obstetric emergencies, medicolegal issues on cesarean section, and video demonstrations of several cases such as breech delivery, twins delivery, external cephalic version, delivery of other abnormal presentations, instrumental delivery, VBAC, and fetal blood sampling. The duration of the 1^st^ day of the course is estimated at approximately 8 h. The 2^nd^ day’s schedule will be mainly devoted to a cardiotocography (CTG) physiological interpretation course (evaluation of fetal distress and avoidance of unnecessary interventions). The duration of the 2^nd^ day of the course is estimated at approximately 6 h.

**Table 4:** HSOG Guidelines and Consent Forms included at the intervention phase (www.hsog.gr)

**Guideline or consent form**	**Title**
Guideline 1	Labor induction
Guideline 2	Cesarean section
Guideline 3	Vaginal birth after cesarean section
Consent form 1	Cesarean section
Consent form 1	Cesarean section for placenta praevia

A hands-on workshop using mannequins will also take place on-site for each unit prior to initiation of the intervention period during the scheduled visit of the members of the Committees and staff: it will include several workstations, such as breech delivery, twins delivery, external cephalic version, delivery of other abnormal presentations, forceps delivery, and VBAC. Participants will have the opportunity to practice what was taught during the previous 2-day theoretical course in a setting simulating real-life conditions. The duration of this activity is estimated at 2–3 h, depending on the number of participants from each unit.

An online cesarean section and CTG meeting with a review of the patient notes will take place once or twice monthly with participants from the units, who will at that same point of time be at the intervention phase of the study.

The online courses and hands-on workshops will be given on consecutive days and will be conducted by experienced trainers. Training groups will be limited to only the participating professionals each time in order to ensure adequate training. The purpose of this activity is to make clear the study’s objectives, which are to focus on the need for evidence-based clinical practice and to review the HSOG Guidelines on labor induction, cesarean section, and vaginal birth after cesarean section, along with HSOG’s consent forms. Another crucial aim is to pinpoint what are the barriers to the use of these guidelines in the participating obstetric departments, overcome these barriers, and eventually implement the guidelines.

5. Throughout the intervention period, compliance with guidelines will be enhanced by regular follow-up meetings with the Principal Investigator, members of the committees, and local principal investigators or opinion leaders participating in the study, via either physical presence, teleconferences, or phone calls. Feedback will also be adjusted independently based on locally preferred routes of better tailoring the intervention to the unit’s daily practice. Additional visits may follow if deemed necessary based on platform statistics.
6. A member of the study’s staff will visit each unit every 3 months. During the visit, she/he will accommodate data acquisition and local participating obstetricians’ familiarity with the study’s platform while also serving the study committees’ actions indicated locally. A number of at least four visits per participating unit is estimated.
7. Use of the Robson 10-group classification criteria embedded in the electronic questionnaire (Table 5) and written feedback on the unit’s cesarean section rates on a regular basis will identify the higher categories that should and may be reduced further.
8. Live real-time statistics will be provided, as demanded by the unit basis, to enable the participating units to see their performance. This type of feedback is expected to improve adherence to the interventions.
9. Cesarean section and CTG interpretation meetings will be held once to twice monthly during which cesarean sections and “abnormal” CTG cases will be discussed. Participants will be the units that are at the intervention phase of the study precisely when the meeting takes place. The members of steering committee and local contributors to the study may actively participate in these meetings by physical presence, teleconference, or phone call. This is expected to further enhance compliance.
10. Reminders about good obstetric practice will be placed in labor wards, staff rooms, and above theater hand wash basins. These will be short printed messages on the importance of improving the rate of vaginal birth offering birth attendants hints to reduce cesarean section rates. The reminder-texts will be planned in accordance with preferred recommendations, carefully considering the defining features and the barriers to the adoption of evidence-based birth practices and to the use of the guidelines in Greece. These reminders will also be sent to the participants by newsletters and mobile sms. Every week they will receive a different reminder. When the existing reminders come to an end, a restatement of the reminders will follow again by newsletter.

**Table 5:** The 10 groups of the Robson Classification used in the ENGAGE Trial (World Health Organization implementation manual)

**Robson classification**	
1	Nulliparous, single cephalic, ≥ 37 weeks, in spontaneous labor
2	Nulliparous, single cephalic, ≥ 37 weeks, induced or cesarean section before labor
3	Multiparous (excluding previous cesarean section), single cephalic, ≥ 37 weeks, in spontaneous labor
4	Multiparous (excluding previous cesarean section), single cephalic, ≥ 37 weeks, induced or cesarean section before labor
5	Previous cesarean section, single cephalic, ≥ 37 weeks
6	All nulliparous breeches
7	All multiparous breeches (including previous cesarean section)
8	All multiple pregnancies (including previous cesarean section)
9	All abnormal lies (including previous cesarean section)
10	All single cephalic, < 37 weeks (including previous cesarean section)

This type of study was chosen as the optimal method to examine the effects of a new intervention applied in several population groups and analyzing/measuring the performance of the intervention for each unit’s population individually and as a whole. As already mentioned, the intervention study is characterized by a random sequel from the observation status to the intervention mode in different time intervals (every month) for each two of the participating units. This will create informative data at each step for both the observation and intervention periods.

### Randomization

The Greek territory has been divided into six geographic divisions based on location. Each of the participating departments will be randomly assigned per stratum to the 11 intervention steps, using randomly generated numbers produced by a blinded statistician. Thus, for example, departments in public hospitals nevertheless will have the same likelihood of initiating interventions at any particular step of the trial. A minimization procedure will be used to ensure that there is an equilibrium between an intervention and an observation phase based on several variables: baseline cesarean section rates, region of Greece, and unit size (annual number of births). Minimization has the advantage of matching small numbers of similar units which are analogous in certain unit characteristics [34, 35].

The totality of participating units will be entering the study simultaneously. An independent data center (Women’s Health Research Institute, University of British Columbia, Department of Obstetrics & Gynecology, Vancouver, Canada) will carry out the analysis of the baseline data as well as the allocation procedure and they will communicate their results to HSOG in Greece. This means that there will be a clear-cut distinction between study coordinators and generator of the intervention allocation [36]. All data and the results of the intervention, which latter will be analyzed in several steps, will be available at any time. Additional data relating to the puerperium period will be collected from women.

### Data

The ideal goal is to accumulate 20,000–25,000 deliveries, the sample size having been estimated based upon hospital birth rates for the year 2019. Based on these data, an assumed 57% cesarean rate (also from 2019 data) across centers, an intracluster correlation (ICC) of 0.3, a negligible secular trend, and a significance level of 0.05, we have > 99% power to detect an 8% absolute reduction in cesarean section rates (OR = 0.72). This calculation is based on the method of Hussey and Hughes, which assumes equal cluster sizes. Given that in this trial there is a large variation in the number of deliveries per hospital this assumption may be violated. To ensure the estimated power is robust to this and across possible randomization sequences, we carried out a simulation study. The parameters for the simulation were the same as those above, other than allowing for varying hospital sizes. The ICC was also varied to assess robustness to higher/lower values. In all scenarios, the available sample size resulted in >95% power. All simulations were conducted using R statistical software.

Intervention is the task of the obstetricians of the participating hospitals, but the analysis and conclusions will be derived from mothers and infants by calculating cesarean section rates and maternal and neonatal morbidity. While our intervention is aimed at introducing modifications and improvements within the national public and private health system, the evaluation will take place on both a local and a national basis.

The data will be collected after the completion of a cesarean section or a vaginal delivery by filling in the study questionnaires, one for the mother and the individual delivery and another for the final check of the newborn prior to discharge. The questionnaire data will be separately filled in for each birth in each maternity hospital during the observation or intervention period. The study questionnaire concerns (a) cesarean section indications, normal delivery, instrumental delivery, induction of labor, etc., and (b) data on maternal and neonatal morbidity and mortality. The questionnaires are uploaded to the trial’s website and they are based on the expected primary and secondary outcomes. A second questionnaire will be completed after contacting the mothers who participated in the study following their puerperium period (40 days or later). The obstetrician or a member of his/her team completes these questionnaires. A telephone call to the woman takes place by the study obstetrician and he/she fills the respective online questionnaires. The continuous data flow of new cases to the obstetricians is intended to assist and stimulate the obstetricians’ smooth transition to an updated mode of thinking and working. Moreover, standard questionnaires have been developed in order to minimize the additional tasks related to data collection carried out by the birth attendants in the course of the trial [37].

The main outcome of the study will be the change in the cesarean section rates and how effective the particular interventions are. The secondary outcomes of the study will be the maternal and neonatal morbidity that has occurred (Tables 6 and 7). The outcome variables will be the percentage of primary and secondary outcomes measured during the intervention period and the 2 months following the end of the intervention (rollout period). The amassed data will be employed in order to examine the potential for confounding of the intervention’s main effect on account of imbalances due to group randomization. There will be a clear separation of the data collection system for the determination of outcomes from the execution of the interventions. It will not be possible to blind the randomization due to the nature of the intervention; thus, data collectors will know whenever they are participating in the intervention. Finally, because the ability of participating hospitals to collect and review clinical data might possibly introduce bias into the outcome assessment, the data collection system will be, as far as possible, isolated from the intervention instruments to minimize the bias.

**Table 6:** Maternal morbidity questionnaire: list of complications, diseases, and operations that will be included in the trial

**Maternal primary outcomes**	**Maternal secondary outcomes**
Manual removal of placenta	PPH (postpartum hemorrhage, secondary)
PPH (postpartum hemorrhage)	Removal of retained products (curettage, medicines)
Tachypnea /Bradypnea	Anemia
Oliguria that does not respond to fluids or diuretics	Blood transfusion
Inability to form clots (clotting disturbances)	Hysterectomy
Shock	Thrombosis/embolism
Cardiac arrest (absence of pulse and loss of consciousness)	Ileus
Cardiopulmonary resuscitation	Necrotizing enterocolitis
Severe acute thrombocytopenia (< 50,000 platelets/mL)	Fistulas
Red blood cell transfusions (≥ 5 units)	Wound dehiscence
ICU (intensive care unit) admission	Trauma infection
3^rd^ or 4^th^ degree perineal tear	Prolonged bladder catheterization
Uterine rupture	Urinary tract infection
Laparotomy (including hysterectomy)	Endometritis
Preeclampsia/eclampsia	Puerperal fever
Stroke	Sepsis
Uncontrolled convulsions/status epillepticus	Pelvic organ prolapse
Permanent neurological injury	Incontinence
Other	Puerperal psychiatric disorders

**Table 7:** Neonatal morbidity, complications, and their monitoring

**Neonatal outcomes**
Breastfeeding issues
Transient tachypnea of the newborn (TTN)
Respiratory distress syndrome (RDS)
Intracranial hemorrhage
Brachial plexus injury
Other neurologic injury
Neonatal sepsis
Neonatal jaundice
X-ray suggestive of alveolar disease
Admission in NICU
Admission for more than 48 h in NICU
Other

A separate questionnaire has been created to calculate professionals’ willingness to change: this will be completed by all participating obstetricians prior to the intervention period. The Head Obstetrician of each unit fills out another questionnaire, his perception being given special attention. Obstetric practice trends may also be affected by the perceptions and routine practice of other medical specialties in close collaboration with the obstetric unit. Thus, two different questionnaires will also be sent to the Head Anesthesiologist and the Head Neonatologist/Pediatrician of each participating hospital (unit).

The REDCap electronic data platform will be accessed by all participating professionals to insert their data. REDCap is a secure web-based application developed to capture online and offline data for clinical research and operations and to build and manage online surveys and databases. The platform is designed to provide a safe environment so that researchers may collect and store highly confidential and sensitive medical information, while access is limited to authorized persons. All participating women will be included in the data analysis.

This is an anonymous study and no personal identifiers will be transmitted. We will not retain any identity information about the participating women and their babies. The data contained in the current study will be kept strictly confidential, while all research records will be stored in a locked file and all electronic information will be coded and secured using a password-protected file. No personal information will be included in any report we may publish which would make possible identification of the obstetricians or women who participated. The participating obstetricians or any of the pregnant women that they care for will be aware that they can decline to take part in the study without affecting their care or the relationship of the pregnant woman to her obstetrician. They will moreover know that they are entitled not to reply to any question and to opt out at any time during the study period. The trial committees will be available to respond to any further queries about the study. Finally, the participating women will be able to access a summary of the study’s anonymous results uploaded on the trial’s webpage after the conclusion of the study. For the obstetricians, this information will be provided with particular details of their unit performance.

### Statistical analysis

All relevant baseline individual and hospital characteristics will be summarized both overall and by trial arm period. The main analysis of the primary outcome will be based on a generalized logistic regression mixed effects model, with covariates for the trial arm and a categorical term for any underlying trend. The primary effect measure will be the odds ratio for the arm estimated from this model. Depending on the data, to increase precision, the primary model may include parametric modeling of any secular trend rather than categorical.

Sensitivity analyses of the primary outcome (and primary model) may include extensions to allow for varying secular trends between clusters (random effect for time) and varying treatment effect across clusters (random interaction between cluster and trial arm). Effect modification by cluster strata (e.g., public/private) may be explored by the inclusion of a fixed effect interaction between cluster strata and arm.

Although not expected, if baseline imbalances are obvious and of clinical relevance between control and intervention periods, the above models may be adjusted for these imbalances via a further sensitivity analysis. Moreover, if any hospitals display non-adherence to the intervention guidelines, a per-protocol analysis may be conducted ensuring proper adjustment for relevant differences between those who adhere and those who do not.

Secondary outcomes of maternal and neonatal morbidity and mortality will be displayed descriptively and, where possible (sample size permitting), analyzed similarly to the primary outcome.

There will be no interim analyses as safety is not a concern. Significance of any difference in the primary outcome is set at 0.05 and at 0.01 for additional secondary outcomes. All analyses will be conducted using R statistical software.

### Trial administration, oversight, and monitoring

The trial will be administered throughout Greece by local research and clinical staff. Database programming, statistical analyses, and research support will be provided by a Clinical Trial Unit at the University of British Columbia, Vancouver, which has a long history of implementing and supporting clinical trials.

Oversight and monitoring are the tasks of the trial steering and executive committees under the feedback of the coordinating center platform. The trial committee members are presented in Table 8. A Data Monitoring Committee (DMC) and a Data Safety Committee, both with no competing interests, will provide an independent assessment of the trial as it is expected to have a major impact on clinical practice.

**Table 8:** The ENGAGE Trial Committees

**Steering committee** Provides oversight and supervision of the conduct of the trial on behalf of the PI and the Sponsor.	Professor Dimitrios Loutradis^a^ (Chair), Professor Nikolaos Vrachnis, Professor Aris Papageorgiou, Professor Peter Von Dadelszen, Professor Laura Magee, Marianne Vidler, Jeffrey Bone, Professor Apostolos Athanasiadis^a^, Dr Christodoulos Akrivis, Professor Aris Antsaklis, Professor Georgios Adonakis, Professor Nikolaos Vlahos^a^, Professor Grigorios Grimbizis, Professor Alexandros Daponte^a^, Professor Peter Drakakis^a^, Dr Vasileios Sioulas, Dr Nikolaos Kambas, Dr Theodoros Katasos, Michail Matalliotakis, Professor Antonios Makrigiannakis, Dr Ilias Katsikis, Professor Nikolaos Nikolettos, Professor Georgios Pados, Professor Minas Paschopoulos, Dr Konstantinos Patsouras, Dr Meni Saklamaki, Professor Soultana Siahanidou, Dr Vasilios Tsitsis
**Executive committee** Concentrates on the progress of the study, adherence to the protocol, and consideration of new information relevant to the research question.	Professor Nikolaos Vrachnis (Chair), Professor Dimitrios Loutradis^a^, Professor Aris Papageorgiou, Professor Peter Von Dadelszen, Professor Efthimios Deligeoroglou, Professor Peter Drakakis^a^, Professor Nicoletta Iacovidou, Professor Laura Magee, Professor Alexandros Rodolakis^a^, Professor Marleen Temmerman, Marianne Vidler
**Data safety and ethics committee** Responsible for scientific and ethical review of the Trial prior to its initiation and monitoring to ensure ethical compliance during the conduct of the Trial.	Anna Pilar Betran Lazaga, Professor Stamatina Illiodromiti, Professor Evangelia Samoli
Budget and finance committee Negotiates, analyzes, recommends, and regularly reviews the financial components of the clinical trial, in collaboration with the Trial’s sponsor.	Professor Dimitrios Loutradis^a^ (Chair), Professor Nikos Vrachnis, Professor Georgios Adonakis, Dr Emmanouil Doulgerakis, Dr Alexander Mortakis, Dr Paraskevas Petropoulos, Professor Nikolaos Vlahos^a^
**Data monitoring committee** Clinicians and biostatisticians appointed to provide assessment of the scientific validity and integrity of clinical trials. Practical aspects on the progress of the study, adherence to the protocol, data acquisition and storage, solving technical issues when arise.	Professor Nikolaos Vrachnis (Chair), Ass. Professor Nikolaos Antonakopoulos, Dr Georgios Maroudias, Dr Nikolaos Loukas, Dr Nikolaos Roussos, Jeffrey Bone, Sandhu Ash, Marianne Vidler, Dr Stefania Kassaris

^a^Board Members of HSOG (sponsor)

In the case of significant protocol amendments, there is a provision to notify the sponsor and funder first, after which the PI will notify the centers and a copy of the revised protocol will be sent to the local PI to add to the Investigator Site File. Any deviations from the protocol will be fully documented using a breach report form. The protocol will then be accordingly updated in the clinical trial registry. Extra care will be given to follow the on-site visits calendar as precisely as possible (Table 9).

There is an estimate of 300,000 euros for the cost of this project. However, budgeting depends on the free work provided. As most authors provided work for free, it is estimated that the final cost will be much less .

**Table 9:** On-site visits calendar and online meetings and courses

Event	Units (as allocated by randomization)	Calendar
On-site visit and hands-on workstation	Units 1 and 2	End of February 2022
On-site visit and hands-on workstation	Units 3 and 4	End of March 2022
On-site visit and hands-on workstation	Units 5 and 6	End of April 2022
On-site visit and hands-on workstation	Units 7 and 8	End of May 2022
On-site visit and hands-on workstation	Units 9 and 10	End of June 2022
On-site visit and hands-on workstation	Units 11 and 12	End of July 2022
On-site visit and hands-on workstation	Units 13 and 14	End of August 2022
On-site visit and hands-on workstation	Units 15 and 16	End of September 2022
On-site visit and hands-on workstation	Units 17 and 18	End of October 2022
On-site visit and hands-on workstation	Units 19 and 20	End of November 2022
On-site visit and hands-on workstation	Units 21 and 22	End of December 2022
2-day online courses	All units in the intervention arm	1 per month for 11 months (11 in total)
CS and CTG meetings	All units in the intervention arm	1–2 per month for 18 months (18–36 in total)

## Discussion

### Strengths

This is the first large-scale prospective study on cesarean sections in Greece and, simultaneously, the first stepped-wedge randomized trial on maternity care in the country. It is at the same time the first randomized trial with a full spectrum of behavioral, educational, and organizational interventions on cesarean sections internationally.

The designed study has several strengths. It will be a stepped-wedge randomized trial that is expected to enroll 20,000–25,000 births. In addition, the participating hospitals will cover the entire Greek territory and will encompass maternity units not only in large maternity hospitals in many cities but also those in smaller regional hospitals. We thus believe that the women included in the study will be highly representative of the population of Greece. Moreover, a stepped-wedge study design rather than a classic randomized controlled trial was selected in order to more precisely evaluate the different levels of implementation as well as the experience of the participating professionals simultaneously with the obtained measurements.

The primary goal is to study the effect of the implementation of interventions on the cesarean section rates in Greece; however, we will also focus on maternal and neonatal morbidity and mortality. Thus, the questionnaires will highlight the potential impact of our interventions on each unit’s outcome. An additional secondary target is to appraise the performance of Greek obstetricians with a view to improving obstetric outcomes: all positive conclusions derived from this trial will help to upgrade obstetric services in Greece in the coming years. The study is expected to yield new insights into the effects, advantages, possibilities, and challenges of the consistent implementation of guidelines and other interventions with regard to the practice of cesarean section in Greece.

Another noteworthy strength of the study is that the data will be collected not only from inpatient records (during hospitalization) but also from follow-up phone calls to the new mothers after puerperium. In this way, we expect to collect the maximum of available information and explore in depth the current and the new status of clinical practice after examining the intervention outcome. The study period is as long as required to satisfy our Steering Committee members, as an adequate study period.

### Limitations

Our study will have a few inevitable limitations. No preliminary pilot study on the subject has taken place, which could have allowed an even better study design, though inevitably a longer preparation. We nevertheless feel that this large pioneer study will serve as an incentive for larger studies in the future.

## Trial status

Protocol version No 6 (15-09-2023). The study is currently at the intervention stage. The recruitment began on the first of June 2021 and is expected to be completed by the last day of October 2023. The trial protocol was submitted for publication one month prior to the last participants’ visits. We initially considered to submit our protocol ahead of this time, but we wanted to be as accurate as possible and our interventions to be reproducible from other researchers in the future, so as to reach the same conclusions provided that all other trial claims in their research are similar to ours. At this stage of our protocol, we don't expect that any part of our interventions will be modified, and we anticipate that all our research findings will be credible for the public health and interest.

There was public or patient involvement in the design of the protocol. Our website *emgetrial.gr* was open to the public and to journalists so that they could send their comments [38, 39].

### Supplementary Information

Additional file 1. Health professional’s consent for participation in the engage study.

Additional file 2. Maternal consent for participation in the engage study.

Additional file 3. 

## Data Availability

The data supporting the findings of the study will be kept by the Principal Investigator and corresponding author Nikolaos Vrachnis and will be available upon reasonable request and with permission of the principal investigator. Site investigators or Steering Committee members do not have access to the full dataset.
