# Supplementary material for: ENhancinG vAGinal dElivery in Greece through educational and behavioral interventions among maternity care providers regarding labor management: the ENGAGE stepped-wedge randomized prospective trial protocol
Source: Trials. 2024 Aug 19;25:548. doi: 10.1186/s13063-024-08263-x (PMC11331648; doi:10.1186/s13063-024-08263-x)
Supplement: Supplementary file 1 — Additional file 1. Health professional’s consent for participation in the engage study. [file 13063_2024_8263_MOESM1_ESM.docx]

**Appendix 1**

**Health professional’s consent for participation in the engage study**

**Title of the study:**

A stepped-wedge randomized controlled intervention trial to enhance vaginal delivery and reduce cesarean sections in Greece

**Principal Investigator:** Professor Nikolaos Vrachnis

**Introduction**

We invite you to participate in the ENGAGE study on the causes and mode of delivery in Greece (vaginal birth or cesarean section). As the clinic where you work participates in the study, you are asked to read the consent and if you agree to sign it. For any question you may address to the local principal investigator, as well as read the detailed protocol and questionnaires of the study that have already been sent to you and your clinic.

**Purpose of the study**

The purpose of the study is to investigate the rates of vaginal delivery and cesarean section in Greece after the implementation of guidelines and other interventions on health professionals of the participating maternity unit. This research study will also gather data related to maternal and neonatal morbidity in Greece.

**Description of the study process**

If you agree to participate in the study, for each delivery you are involved in, you must complete the respective study questionnaires depending on your specialty. The questionnaires concern pregnancy, labor and any morbidity of the mother and the newborn, both directly after birth and 1-3 months after birth (short telephone communication with the mother).

**Confidentiality**

This study is anonymous. We do not store any information about your identity. There is no correlation between the health professional and the women participating in the study. The files with the study data will remain strictly confidential and will be kept locked in a restricted and code-protected database. Also, no personal information will be included in the reports or scientific articles that will be published. You will have access to your unit data and anonymous data from other participating units.

**Payment**

Your participation is voluntary, and you will not receive any payment / compensation.

**Security**

Participation in the study does not pose any risk to women and their babies because no new drugs are being tested. Furthermore, the application of the study interventions is on your choice.

**Right of refusal or withdrawal**

The decision to participate in this study is entirely up to you. You can refuse to take part in the study and this decision will not affect your position in the clinic. You have the right to withdraw from the study at any point during the process.

**Right to ask questions**

You have the right to ask questions about the study and receive answers to your questions before, during or after the study. If you have further questions about the study, you can contact the local principal investigator or the members of the study’s scientific committee at any time.

**Consent**

Your signature indicates that you have decided to participate voluntarily in this study and that you have read and understood the information provided above. We may provide a copy of this consent form upon your request.

**DECLARATION OF CONSENT**

In view of the above information and what has been explained to me by the local principal investigator, I give my consent and agree to participate in the ENGAGE trial with the above title. The clinic I work in participates in the trial after approval by the scientific committee of the hospital. I declare that I will contribute to the study providing the necessary information as included in the study’s questionnaires, concerning details of the entire maternal and neonatal management and treatment, as well as the early and late outcomes.

Finally, I declare that I will obtain an informed consent, as provided by HSOG, from the participating women regarding the provision of medical information of themselves and their babies.

The health professional

Signature:

Full name:

Specialty: (Obstetrician, Pediatrician, Midwife)

Unit/Hospital:

Date:
