# Supplementary material for: ENhancinG vAGinal dElivery in Greece through educational and behavioral interventions among maternity care providers regarding labor management: the ENGAGE stepped-wedge randomized prospective trial protocol
Source: Trials. 2024 Aug 19;25:548. doi: 10.1186/s13063-024-08263-x (PMC11331648; doi:10.1186/s13063-024-08263-x)
Supplement: Supplementary file 2 — Additional file 2. Maternal consent for participation in the engage study. [file 13063_2024_8263_MOESM2_ESM.docx]

**Appendix 2**

**Maternal consent for participation in the engage study**

**Title of the study:**

A stepped-wedge randomized controlled intervention trial to enhance vaginal delivery and reduce cesarean sections in Greece

**Principal Investigator:** Professor Nikolaos Vrachnis

**Introduction**

We invite you to participate in a clinical trial on the causes and the mode of delivery in Greece (vaginal delivery or cesarean section). You have been selected as a possible participant as the maternity unit where you will give birth participates in the study. Please read this form and ask any questions before agreeing to participate in the study.

**Purpose of the study**

The purpose of the study is to investigate the rates of vaginal delivery and cesarean section in Greece after the implementation of new guidelines and other interventions to the staff of the participating clinic. These interventions do not concern you or your baby. Even more, this study is expected to reveal possible ways to reduce maternal and neonatal morbidity in Greece.

**Description of the study process**

If you agree to participate in the study, the Obstetrician responsible for your labor will fill in a questionnaire concerning your pregnancy, labor and postpartum course. In addition, the pediatrician or midwife will fill in a questionnaire concerning your baby. Finally, 1-3 months after your labor you will receive a phone call from our team to answer some questions about you and your baby concerning the period after hospoital discharge. The phone call will not take more than 5 minutes.

**Risks / difficulties that will arise from the study**

There are no foreseeable (or expected) risks to you or your family.

**Confidentiality**

This study is anonymous. We will not retain any identity information. Study records will remain strictly confidential. The files will be kept in a locked file and all electronic data will be encrypted and protected in a password file. We will not include any of your personal details in any report or scientific article published. Only anonymized final results will be published and posted on the ENGAGE websites.

**Payment**

**Y**ou will not receive any payment / compensation.

**Right of refusal or withdrawal**

The decision to participate in this study is entirely up to you. You can refuse to take part in the study and this decision will not affect your care. You have the right to refuse to answer questions when we will call you after birth, and to withdraw from the study at any point. In addition, you have the right to request from the research team not to use any of your data.

**Right to ask questions**

You have the right to ask questions about the study and receive answers before, during or after the study. If you have further questions about the study, you can contact the doctor responsible for your delivery at any time. If you wish so, we will provide you access to a summary of the study results after its completion, on the ENGAGE website.

**Consent**

Your signature indicates that you have decided to participate voluntarily in this study and that you have read and understood the information provided above. You will be given a copy of this consent form if you wish.

**Declaration of consent**

In view of the above information and what has been explained to me, I give my consent and agree to participate in the ENGAGE trial with the above title.

Pregnant woman’s Name:

Pregnant woman’s Signature:

Date:

Obstetrician Signature:

Date:
